# Associations between Dietary Polyphenols and Type 2 Diabetes in a Cross-Sectional Analysis of the PREDIMED-Plus Trial: Role of Body Mass Index and Sex

**DOI:** 10.3390/antiox8110537

**Published:** 2019-11-08

**Authors:** Anna Tresserra-Rimbau, Sara Castro-Barquero, Facundo Vitelli-Storelli, Nerea Becerra-Tomas, Zenaida Vázquez-Ruiz, Andrés Díaz-López, Dolores Corella, Olga Castañer, Dora Romaguera, Jesús Vioque, Ángel María Alonso-Gómez, Julia Wärnberg, José Alfredo Martínez, Lluís Serra-Majem, Ramon Estruch, Francisco José Tinahones, José Lapetra, Xavier Pintó, Josep Antoni Tur, José López-Miranda, Laura García-Molina, Miguel Delgado-Rodríguez, Pilar Matía-Martín, Lidia Daimiel, María Rubín-García, Josep Vidal, Alba Galdon, Emilio Ros, Francisco Javier Basterra-Gortari, Nancy Babio, José Vicente Sorlí, Álvaro Hernáez, Jadwiga Konieczna, Leyre Notario-Barandiaran, Lucas Tojal-Sierra, Jessica Pérez-López, Itziar Abete, Jacqueline Álvarez-Pérez, José Carlos Fernández-García, José Manuel Santos-Lozano, Ana Galera-Cusí, Alicia Julibert, Miguel Ruiz-Canela, Raul Martinez-Lacruz, Karla-Alejandra Pérez-Vega, Aina María Galmes-Panades, Concepción Pastor-Polo, Anai Moreno-Rodriguez, Alfredo Gea, Montserrat Fitó, Rosa María Lamuela-Raventós, Jordi Salas-Salvadó

**Affiliations:** 1 Centro de Investigación Biomédica en Red Fisiopatología de la Obesidad y la Nutrición (CIBEROBN), Institute of Health Carlos III, 28029 Madrid, Spain; 2 Unitat de Nutrició, Departament de Bioquímica i Biotecnologia, Universitat Rovira i Virgili, 43204 Reus, Spain; 3 Nutrition Unit, University Hospital of Sant Joan de Reus, 43201 Reus, Spain; 4 Institut d’Investigació Sanitària Pere Virgili (IISPV), 43201 Reus, Spain; 5 Department of Medicine, Faculty of Medicine and Health Sciences, University of Barcelona, 08036 Barcelona, Spain; 6 Institute of Biomedicine (IBIOMED), University of León, 24071 León, Spain; 7 Department of Preventive Medicine and Public Health, University of Navarra, IDISNA, 31008 Pamplona, Spain; 8 Department of Preventive Medicine, University of Valencia, 46010 Valencia, Spain; 9 Cardiovascular Risk and Nutrition Group, Institut Hospital del Mar d’Investigacions Mèdiques (IMIM), 08007 Barcelona, Spain; 10 Health Research Institute of the Balearic Islands (IdISBa), 07120 Palma de Mallorca, Spain; 11 CIBER de Epidemiología y Salud Pública (CIBERESP), Instituto de Salud Carlos III, Madrid, Spain; 12 Miguel Hernandez University, ISABIAL-FISABIO, 03010 Alicante, Spain; 13 Bioaraba Health Research Institute; Osakidetza Basque Health Service, Araba University Hospital; University of the Basque Country UPV/EHU; 01009 Vitoria-Gasteiz, Spain; 14 Department of Nursing. Institute of Biomedical Research in Málaga (IBIMA), University of Málaga, 29010 Málaga, Spain; 15 Department of Nutrition, Food Sciences, and Physiology, Center for Nutrition Research, University of Navarra, 31008 Pamplona, Spain; 16 Nutritional Genomics and Epigenomics Group, IMDEA Food, CEI UAM + CSIC, 28049 Madrid, Spain; 17 Research Institute of Biomedical and Health Sciences (IUIBS), University of Las Palmas de Gran Canaria & Centro Hospitalario Universitario Insular Materno Infantil (CHUIMI), Canarian Health Service, 35016 Las Palmas de Gran Canaria, Spain; 18 Department of Internal Medicine, Institut d’Investigacions Biomèdiques August Pi Sunyer (IDIBAPS), Hospital Clínic, University of Barcelona, 08036 Barcelona, Spain; 19 Virgen de la Victoria Hospital, Department of Endocrinology, Instituto de Investigación Biomédica de Málaga (IBIMA). University of Málaga, 29010 Málaga, Spain; 20 Department of Family Medicine, Research Unit, Distrito Sanitario Atención Primaria Sevilla, 41010 Sevilla, Spain; 21 Lipids and Vascular Risk Unit, Internal Medicine, Hospital Universitario de Bellvitge-Idibell-Universitat de Barcelona, 08908 Hospitalet de Llobregat, Spain; 22 Research Group on Community Nutrition and Oxidative Stress, University of Balearic Islands, 07122 Palma de Mallorca, Spain; 23 Department of Internal Medicine, Maimonides Biomedical Research Institute of Cordoba (IMIBIC), Reina Sofia University Hospital, University of Cordoba, 14004 Cordoba, Spain; 24 Department of Preventive Medicine and Public Health, University of Granada, 18016 Granada, Spain; 25 Division of Preventive Medicine, Faculty of Medicine, University of Jaén, 23071 Jaén, Spain; 26 Department of Endocrinology and Nutrition, Instituto de Investigación Sanitaria Hospital Clínico San Carlos (IdISSC), 28040 Madrid, Spain; 27 CIBER Diabetes y Enfermedades Metabólicas (CIBERDEM), Instituto de Salud Carlos III (ISCIII), 28029 Madrid, Spain; 28 Department of Endocrinology, Institut d’Investigacions Biomédiques August Pi Sunyer (IDIBAPS), Hospital Clínic, University of Barcelona, 08036 Barcelona, Spain; 29 Hospital Universitario Fundación Jimenez Díaz, 28040 Madrid, Spain; 30 Lipid Clinic, Department of Endocrinology and Nutrition, Institut d’Investigacions Biomèdiques August Pi Sunyer (IDIBAPS), Hospital Clínic, 08036 Barcelona, Spain; 31 Department of Internal Medicine (Endocrinology), Hospital Reina Sofía, 31500 Tudela, Spain; 32 Centro Salud Raval-Elche, 03203 Alicante; 33 Department of Nutrition, Food Science and Gastronomy, XaRTA, INSA, School of Pharmacy and Food Sciences, University of Barcelona, 08028 Barcelona, Spain

**Keywords:** diet, obesity, flavonoids, catechins, proanthocyanidins, hydroxybenzoic acids, hydroxycinnamic acids, lignans, phenolic acids

## Abstract

Overweight and obesity are important risk factors for type 2 diabetes (T2D). Moving towards healthier diets, namely, diets rich in bioactive compounds, could decrease the odds of suffering T2D. However, those individuals with high body mass index (BMI) may have altered absorption or metabolism of some nutrients and dietary components, including polyphenols. Therefore, we aimed to assess whether high intakes of some classes of polyphenols are associated with T2D in a population with metabolic syndrome and how these associations depend on BMI and sex. This baseline cross-sectional analysis includes 6633 participants from the PREDIMED-Plus trial. Polyphenol intakes were calculated from food frequency questionnaires (FFQ). Cox regression models with constant time at risk and robust variance estimators were used to estimate the prevalence ratios (PRs) for polyphenol intake and T2D prevalence using the lowest quartile as the reference group. Analyses were stratified by sex and BMI groups (overweight and obese) to evaluate potential effect modification. Catechins, proanthocyanidins, hydroxybenzoic acids, and lignans were inversely associated with T2D. Hydroxycinnamic acids were directly related in men. These associations were different depending on sex and BMI, that is, women and overweight obtained stronger inverse associations.

## 1. Introduction

The number of people with diabetes has been increasing over the past few decades and the estimations for the near future are not encouraging. Currently, 8.5% of adults have diabetes worldwide, type 2 diabetes (T2D) being the most prevalent type. People with T2D have increased odds of premature death and are more likely to have myocardial infarction or angina, stroke, kidney failure, peripheral artery disease, vision loss, and neuropathy. Therefore, T2D may be considered a global public health problem with vast economic consequences [1].

Unlike type 1 diabetes, T2D can be prevented through different approaches, namely, exercising regularly, avoiding smoking, keeping weight under control, and adhering to a healthy diet. There is compelling evidence from prospective observational studies and clinical trials that individual nutrients, foods, and dietary patterns are crucial in the prevention and management of T2D. It is well known that plant-based diets including whole grains, fruits, vegetables, legumes, and nuts as well as moderate alcohol consumption have been associated with lowering the risk of T2D, whereas diets rich in refined grains, red or processed meats, and sugar-sweetened beverages increase the risk [2]. Plant foods are usually rich in polyphenols, a large and heterogeneous group of bioactive compounds that constitute the first source of antioxidants in the diet. Polyphenols are usually classified according to their structure into two main groups—flavonoids and non-flavonoids. The flavonoid group, with C6–C3–C6 structured compounds, includes flavones, flavonols, theaflavins, catechins, proanthocyanidins (polymeric forms), flavanones, anthocyanidins, and isoflavones. Phenolic acids, lignans, and stilbenes belong to the non-flavonoid group. High polyphenol intake has been associated with a reduced incidence of T2D in epidemiological studies [3], and better metabolic control in those participants with T2D. Some of the mechanisms by which dietary polyphenols and their metabolites exert their effects have been elucidated but others are still unknown [4].

Overweight and obese individuals are at higher risk of T2D than lean ones, therefore it seems that they would particularly benefit from polyphenol intake. However, some literature suggest that those with higher body mass index (BMI) may have altered absorption or metabolism of some nutrients and dietary components, including polyphenols [5,6].

Due to the heterogeneity of polyphenols, and to understand their antidiabetic properties, it is necessary to study them considering the different groups separately, because they have different absorption and metabolism [7]. Epidemiological and clinical studies have usually been focused on some polyphenolic groups, but comprehensive studies are still scarce. Moreover, to our knowledge, no previous studies have examined the associations between the intake of all polyphenolic groups and subgroups and the risk of T2D stratifying by sex and BMI. Therefore, our aim was to examine the hypothesis that higher intakes of some classes of polyphenols are associated with T2D in a population with metabolic syndrome and these associations depend on sex and BMI.

## 2. Materials and Methods

### 2.1. Study Design and Population

The present study was designed as a baseline cross-sectional analysis in the PREDIMED-Plus trial, a six-year parallel-group, multicenter, randomized, lifestyle intervention study for the primary prevention of cardiovascular disease involving 6874 participants recruited in 23 Spanish recruiting centers from October 2013 to December 2016. Eligible participants were men (aged 55–75 years) and women (aged 60–75 years) with a BMI between 27 and 40 kg/m^2^ who met, at least, three components of the metabolic syndrome (updated harmonized criteria of the International Diabetes Federation and the American Heart Association and National Heart, Lung, and Blood Institute).

Details on the PREDIMED-Plus protocol can be found at http://predimedplus.com. The selection and the description of the studied sample have been previously reported [8]. The PREDIMED-Plus trial was registered at the International Standard Randomized Controlled Trial (ISRCTN89898870; registration date, 24 July 2014). All participants provided written informed consent, and the study protocol and procedures were approved according to the ethical standards of the Declaration of Helsinki by all the participating institutions: CEI Provincial de Málaga, CEI de los Hospitales Universitarios Virgen Macarena y Virgen del Rocío, CEI de la Universidad de Navarra, CEI de las Illes Balears, CEIC del Hospital Clínic de Barcelona, CEIC del Parc de Salut Mar, CEIC del Hospital Universitari Sant Joan de Reus, CEI del Hospital Universitario San Cecilio, CEIC de la Fundación Jiménez Díaz, CEIC Euskadi, CEI en Humanos de la Universidad de Valencia, CEIC del Hospital Universitario de Gran Canaria Doctor Negrín, CEIC del Hospital Universitario de Bellvitge, CEI de Córdoba, CEI de Instituto Madrileño De Estudios Avanzados, CEIC del Hospital Clínico San Carlos, CEI Provincial de Málaga, CEI de las Illes Balears, CCEI de la Investigación Biomédica de Andalucía and CEIC de León. The code of the Etical Committe aproval of the Coordinated Center (CEIC del Hospital Universitari Sant Joan de Reus) is 13-07-25/7proj2 (aproval date: 30/07/2013).

After the exclusion of 53 participants with missing baseline dietary data and 188 with extreme energy intakes (<500 or >3500 for women and <800 and >4000 for men) [9], 6633 participants were available for the present analysis.

### 2.2. Dietary Assessment and Polyphenol Intake

At baseline, participants filled out a validated 143-item semi-quantitative food frequency questionnaire (FFQ) [10] under the supervision of registered dietitians, from which total energy and nutrient intake were calculated on the basis of Spanish food composition tables [11,12]. Additionally, they were asked to fill out a 17-point score questionnaire measuring adherence to an energy-restricted traditional Mediterranean diet (MedDiet), an updated version of the 14-point score questionnaire used in the PREDIMED trial [13].

The 143-item FFQ was also used to calculate polyphenol intake together with the Phenol-Explorer database (www.phenol-explorer.eu). The validity of this particular FFQ to assess total polyphenol intake was tested in both clinical and cross-sectional studies [14,15]. Individual polyphenol intakes were obtained by multiplying the content of each polyphenol in each food item with polyphenols (mg/g) by the daily consumption of this food item (g/day) and then summing the product across all food items. Then, polyphenol subclasses were adjusted for total energy intake using the residual method [9]. After stratification by sex and BMI groups, namely, overweight (BMI < 30 kg/m^2^) and obese (BMI ≥ 30 kg/m^2^), polyphenol intakes were divided in quartiles.

### 2.3. Ascertainment of T2D

For the present analysis, the main endpoint was the prevalence of T2D, defined as previous clinical diagnosis of T2D, or glycated hemoglobin (HbA1c) ≥ 6.5%, or use of antidiabetic medication at baseline, or fasting plasma glucose > 126 mg/dl in both the screening visit and baseline visit.

### 2.4. Assessment of Covariates

Participants filled out a general questionnaire to provide data on lifestyle habits, education, concurrent diseases, and medication use. Physical activity was measured by a short Minnesota Leisure Time Physical Activity Questionnaire validated for the Spanish population [16]. Height and weight were measured by trained staff. BMI was calculated as weight in kilograms by the square of height in meters. After an overnight fasting, blood samples were collected. Serum glucose, triglyceride, as well as total and high-density lipoprotein (HDL) cholesterol levels were measured by routine laboratory tests using standard enzymatic methods.

### 2.5. Statistical Methods

Baseline characteristics according to quartiles of total polyphenol intake are presented as means (±SD) for quantitative variables and frequencies for categorical variables. One-factor ANOVA tests were used to assess the differences between quartiles of polyphenol consumption and chi-squared tests for categorical variables.

We used Cox regression models with constant time at risk and robust variance estimators to obtain the prevalence ratios (PRs) for polyphenol intake and T2D prevalence using the lowest quartile as the reference group [17]. We ran stratified analyses for sex and BMI groups (overweight and obese) to evaluate potential effect modification.

In multivariate models, we adjusted for age (continuous), smoking status (never, current, or former), physical activity (quintiles), and education (primary education, secondary education, academic/graduate) in model 1. In the fully adjusted model, we additionally adjusted for energy intake (continuous) as well as consumption of refined cereals and animal products (derived from the 17-point questionnaire) and of distilled beverages and liquors, sugar, soft drinks, cookies, and pastries (in g/day derived from the FFQ). All models were stratified by the recruiting center.

Interaction effect was calculated comparing the models, with and without the interaction term, with likelihood-ratio tests after estimation. We tested for linear trends across categories of polyphenol intake by assigning each participant the median value for each category and modelling this value as a continuous variable. Statistical analyses were conducted using STATA software (version 15.1; StataCorp, College Station, TX, USA). All *t*-tests were two-sided and *p*-values below 0.05 were considered significant.

## 3. Results

The present study was conducted on 6633 participants from the PREDIMED-Plus cohort, 51.6% men, with a mean age of 65.0 ± 4.9 years. Of these, 2042 had T2D at baseline (30.8%). Mean total polyphenol intake was 846 ± 275 mg/day. Approximately 58% corresponded to flavonoids, 33% were phenolic acids, and the rest were stilbenes, lignans, and other polyphenols. The flavonoid group was mainly composed of proanthocyanidins (205 ± 175 mg/day), flavanones (86 ± 75 mg/day), flavones (72 ± 45 mg/day), flavonols (54 ± 22 mg/day), anthocyanidins (44 ± 36 mg/day), and catechins (28 ± 22 mg/day). Other flavonoid subclasses, namely, chalcones, dihydrochalcones and dihydroflavonols, theaflavins, and isoflavonoids, contributed less than 1% and were not considered in the analyses. Within phenolic acids, hydroxycinnamic acids were the main subclass (>90%) and hydroxybenzoic acids followed them with 6%. Other minor phenolic acids were also not considered. Intake of energy-adjusted polyphenols by sex and BMI group is shown in Table S1, Supplementary Materials. Men tended to have higher intakes of total polyphenols and total phenolic acids, as well as anthocyanidins, catechins, hydroxycinnamic and hydroxybenzoic acids, stilbenes, and lignans, whereas women had higher intakes of flavones. Considering BMI groups, overweight men had higher intakes of total flavonoids and proanthocyanidins, but lower intakes of total phenolic acids and hydroxycinnamic acids compared to obese men. On the other hand, women within the overweight group had higher intakes of hydroxybenzoic acids and stilbenes but lower intakes of hydroxycinnamic acids.

Table 1 shows baseline participant characteristics by quartiles of energy-adjusted total polyphenol intake. At baseline, participants in the highest quartile were more likely to be men, physically active, smokers and former smokers, and with a higher education level. The same characteristics are summarized according to sex and BMI groups in Table S2, Supplementary Materials. In this cohort, men were younger (because of the inclusion criteria), had higher BMI, were more physically active, and were more highly educated than women. Within the group of men, there were more smokers, the prevalence of T2D was higher, as well of T2D treatment users. Their fasting glucose levels were more elevated, but not HbA1c. Overweight men and women had lower prevalence of T2D (although not significant in the case of men), lower fasting glucose levels, were more physically active, and were more educated. Moreover, overweight men were older, and overweight women used less metformin, had lower levels of HbA1c, and smoked more.

In Table 2, we summarize nutrient and main food group consumption according to energy-adjusted quartiles of polyphenol intake. Those in the fourth quartile tended to have a healthier diet in general, with higher consumption of fruits, vegetables, and nuts, and lower consumption of cereals, dairy products, meat, and sugar. They also presented higher intake of total carbohydrates and fiber, and lower intake of fatty acids compared to those in the reference quartile. However, they also had greater intake of alcohol, soft drinks, and cookies, pastries, and sweets, and lower consumption of olive oil. Nutrient composition of the diet and main food items according to sex and BMI groups are presented in Table S3, Supplementary Materials. There were no significant differences in total caloric intake between overweight and obese, men or women. Generally, women had healthier diets (higher score in the 17-point MedDiet questionnaire), with greater consumption of fiber, vegetables, dairies, and fish, and less alcohol. No differences were observed for legumes and nuts between sex. Obese men also reported to consume more meat, but less sugar than overweight men. On the other hand, obese women had poorer Mediterranean diet adherence, with less consumption of polyunsaturated fatty acids (PUFAs) and nuts, and less alcohol.

**Figure 1:**
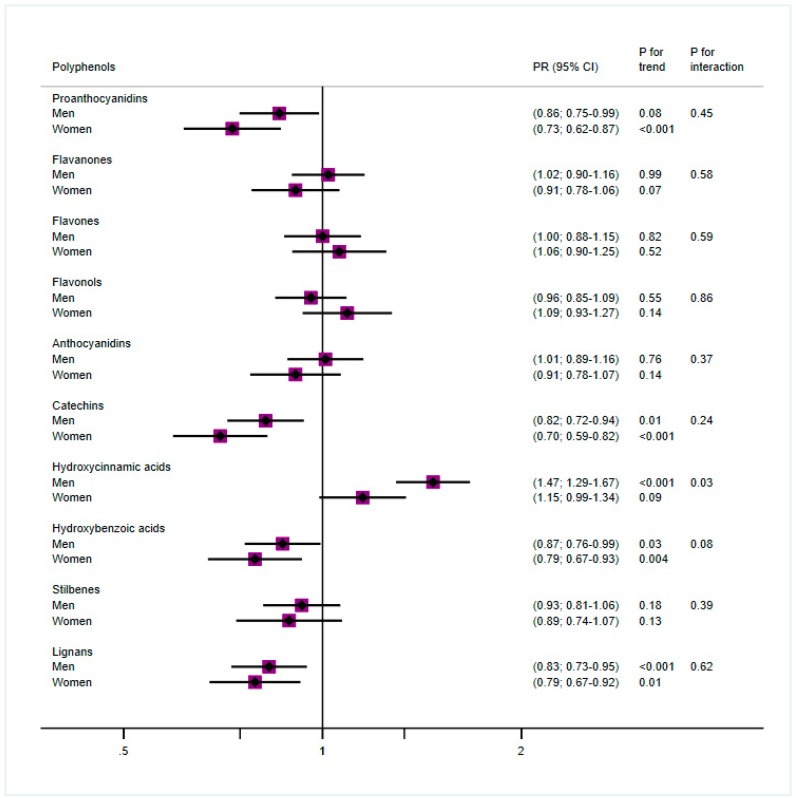
Cox proportional models for polyphenol groups and type 2 diabetes (T2D) by sex, comparing quartile 4 (Q4) vs. quartile 1 (Q1) and adjusted for age, education (basic studies, medium, high), body mass index (BMI) (overweight, obese), smoking (never, former, smoker), physical activity (quintiles), energy, consumption of refined cereals and animal products (from 17-point questionnaire) and of distilled beverages and liquors, sugar, soft drinks, cookies, and pastries (g/day), and stratified by recruitment center.

We performed Cox proportional models with constant time to study the association between T2D prevalence and quartiles of the following main polyphenol groups: flavonoids (proanthocyanidins, flavanones, flavones, flavonols, anthocyanidins, and catechins), phenolic acids (hydroxycinnamic and hydroxybenzoic acids), stilbenes, and lignans. The results of the fully adjusted model comparing the fourth versus the first quartiles for men and women are shown in Figure 1. Significant inverse and linear associations were found for catechins, hydroxybenzoic acids, and lignans for both men and women. Proanthocyanidin intake was also inversely associated with T2D, but in men the linearity was not significant. These associations were always stronger for women. On the other hand, hydroxycinnamic acids showed a strong direct association with T2D prevalence in men, but marginally significant in the case of women.

Because weight is an important risk factor for T2D, we also performed the same analysis but dividing the population in two groups according to the BMI—overweight (BMI < 30 kg/m^2^), and obese (BMI ≥ 30 kg/m^2^). In our cohort, three out of four participants were obese. The prevalence of T2D in the groups with obesity compared to the overweight groups was higher in both men (34% vs. 32%) and women (29% vs. 24%).

**Figure 2:**
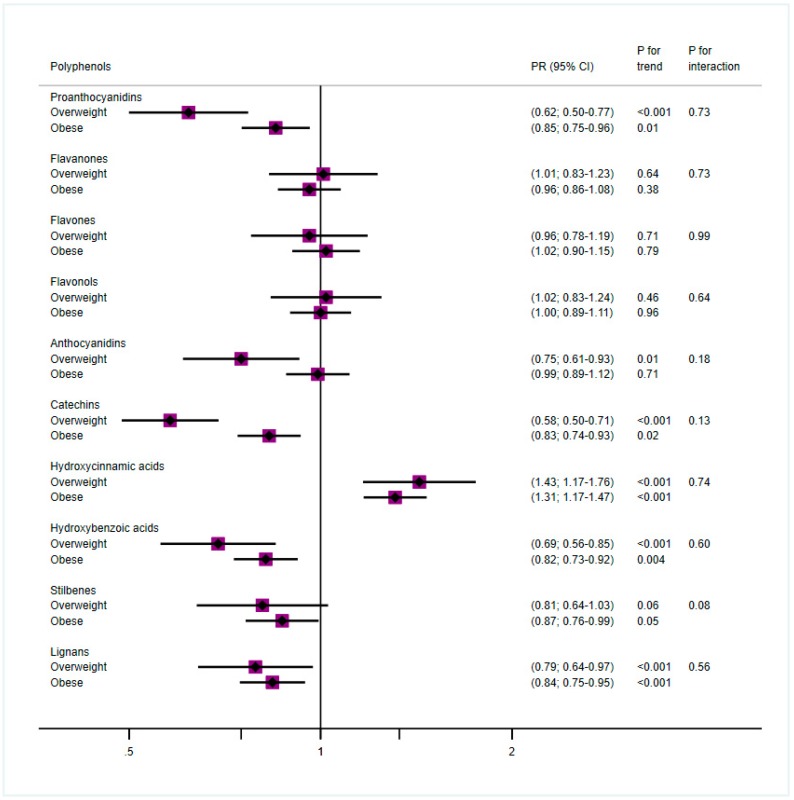
Cox proportional models for polyphenol groups and T2D by BMI groups, comparing Q4 vs. Q1 and adjusted for sex, age, education (basic studies, medium, high), smoking (never, former, smoker), physical activity (quintiles), energy, consumption of refined cereals and animal products (from 17-point questionnaire) and of distilled beverages and liquors, sugar, soft drinks, cookies, and pastries (g/day), and stratified by recruitment center.

Figure 2 shows the results after adjustment for anthropometric, sociodemographic, lifestyle, and dietary variables (fully adjusted model) and stratifying by recruitment center, comparing the fourth versus the first quartiles separated by BMI groups. Significant inverse and linear associations were found for proanthocyanidins, anthocyanidins (only in overweight), catechins, hydroxybenzoic acids, stilbenes (only in obese), and lignans. In all of them, the PR was lower in overweight than in obese. Hydroxycinnamic acid intake was directly associated with T2D in both groups.

Results of the fully adjusted model according to sex and BMI groups are shown in Table 3. We found similar patterns for proanthocyanidins, catechins, and hydroxybenzoic acids. In all cases, there were significant and linear inverse associations comparing extreme quartiles for overweight men and women, and for obese women, but not for obese men. The prevalence ratios were lower for the overweight than the obese. Regarding lignans, inverse associations were significant for all groups except for overweight women. Additionally, we found a significant and inverse linear trend for anthocyanidins and stilbenes in overweight men, although the PR did not reach significance.

Hydroxycinnamic acids, however, were directly associated with T2D in both overweight and obese men, showing a significant linear trend. Non-significant results were found for other polyphenol groups, namely, flavanones, flavones, and flavonols, after adjusting for all potential confounders. Nevertheless, it is worth mentioning that the intake of flavones was directly associated with T2D in men in model 2, prior to adjustment for energy and dietary variables.

## 4. Discussion

In this baseline cross-sectional study within the PREDIMED-Plus trial, we observed that higher intakes of some polyphenolic classes were inversely and lineally associated with the prevalence of T2D in a population at high cardiovascular risk, and these associations depended on sex and BMI. We also found that hydroxycinnamic acids were directly associated with T2D.

Previous epidemiological studies have investigated the association between intake of polyphenols and T2D, but this is the first study that quantifies the association between the intake of different polyphenol subgroups and T2D accounting for differences by sex and BMI.

In our study, catechins were the main source of flavan-3-ols because theaflavin intake was very low. Together with proanthocyanidins, these compounds are classified as flavanols. The main sources of flavanols in the PREDIMED-Plus cohort were cocoa and chocolate, apples, plums, red wine, and tea. Proanthocyanidins and catechins were strongly and inversely associated with T2D in overweight men and women, and obese women. In a previous longitudinal study conducted in a similar cohort (PREDIMED study), proanthocyanidins and catechins showed the same association with new-onset T2D when baseline glucose levels were taken out of the model but not in the fully adjusted model. In a large, prospective, case-cohort study, Zamora-Ros et al. concluded that all flavan-3-ol monomers, including catechins, as well as proanthocyanidins of low polymerization degree were associated with a lower risk of developing T2D. As in our case, they did not find associations for flavonols, except for myricetin intake [18].

Many studies have focused on other monomers of flavan-3-ols, especially epigallocatechin gallate, usually found in tea, grapes, and some seeds, and chocolate catechins. The antidiabetic effects of flavan-3-ols have been reported in animal and cell-culture studies, as well as several clinical studies [4,19,20]. Data from a recent meta-analysis on cocoa intake and cardio-metabolic risk suggested significant improvements on insulin-related outcomes [21].

Anthocyanidins are the blue, red, and purple pigments of most fruits and vegetables, such as berries and wine. Although we only found an inverse linear trend with T2D in overweight men, results from in vitro, animal, epidemiological, and human studies have suggested that anthocyanidins might play an important role modulating T2D [20], even though they have low bioavailability. In the frame of the EPIC-InterAct study, Zamora-Ros et al. did not find a significant association between dietary anthocyanidins and incident T2D, which is in agreement with our findings [22]. However, Jennings et al. found that higher anthocyanin and flavone intake, as well as anthocyanin-rich food consumption, were associated with improvements of insulin resistance in a cross-sectional study with women [23], and higher intakes of these colored compounds were also inversely associated with a lower risk of T2D in the Nurses’ Health Study [24].

Hydroxybenzoic acids followed the same pattern as that of proanthocyanidins and catechins, showing inverse associations in overweight men and women but not in obese men. Red wine, olives, and walnuts were their principal sources in this cohort. Perhaps because they are the minor components of the phenolic acid group, they have not received much attention in previous studies. Hydroxycinnamic acids, on the other hand, accounted for more than 90% of the phenolic acid group, coffee being the main food source. In our cohort, hydroxycinnamic acid intake was associated with higher prevalence of T2D in men and almost in women. This result contradicts other studies regarding coffee and their polyphenols, such as caffeic acid, or chlorogenic acid. Large observational studies have pointed towards a significant inverse association between coffee consumption and T2D, especially with decaffeinated coffee [25,26]. In a double-blind, placebo-controlled, cross-over design, Lane et al. concluded that caffeine impaired postprandial glucose metabolism, especially when caffeine was ingested with carbohydrates. These results differ from those obtained with healthy subjects, suggesting that caffeine, but not other compounds of coffee, could have negative consequences in glucose homeostasis only in diabetics [27]. Our results support the notion that high caffeine intake could be directly associated with T2D.

Stilbenes are non-flavonoid polyphenols, usually known due to its main representative—resveratrol. Red wine is by far the main source of this group of compounds, accounting for more than 90% of the intake. Stilbenes were strongly associated with lower odds of developing T2D in the PREDIMED study [28]; however, we only found a mild association and a linear trend for overweight man in the present cohort. It is worth mentioning that the intake of stilbenes was three times higher in men than in women. More than 50% of women had less than 0.1 mg/day of stilbenes. Clinical and animal models have described the antidiabetic effects of stilbenes [29,30]. Nevertheless, whether stilbenes are feasible for the prevention and/or management of T2D is still controversial [31].

Lignans are phytoestrogens usually found in fiber-rich foods since they are constituents of plant cell walls. In western populations, the consumption of lignans are usually greater than isoflavones. The urinary excretion of lignan metabolites produced by the microbiota (mainly enterodiol and enterolactone) is a marker for fiber intake and whole-grain products [32]. In our population, the main source of lignans was virgin olive oil. Those in the highest quartile of lignan intake had lower odds of having T2D except for overweight women where no association was found. These results agree with the inverse association found a few years ago in the PREDIMED cohort [28]. Also in the same line, lower T2D incidence was found in U.S. women with higher enterodiol and enterolactone in urine, which are gut microbiota metabolites of dietary lignans [33]. However, Zamora-Ros et al. found no significant associations between lignans and T2D incidence in the EPIC study [22].

We did not find significant associations with T2D prevalence for any of the following groups: flavanones, flavones, and flavonols, although in a similar population, flavanones were inversely associated with T2D incidence [28]. In a prospective study conducted in two female large cohorts, urinary excretion of hesperetin (a flavanone) was related to a decreased risk of T2D after a long follow-up. Other polyphenol metabolites, including naringenin, quercetin, isorhamnetin, and caffeic acid, were only inversely associated in the short term [34]. Two large observational studies also support the antidiabetic effects of flavonols [22,35].

We noticed that, in our population, prevalence ratios in overweight participants were generally lower than in obese ones. These results agree with the fact that obesity is associated with lower nutrient bioavailability. Novotny et al. conducted a pilot study to investigate the pharmacokinetic response of grape polyphenols in overweight and obese vs. lean volunteers. They found higher concentrations for catechin, epicatechin, quercetin, and resveratrol in individuals with normal BMI, suggesting that obesity could compromise polyphenol absorption [6]. A previous randomized crossover study with blackberries also reported that lean volunteers had better anthocyanin metabolism than overweight/obese ones [5].

The exact mechanisms by which polyphenols affect T2D are still unknown. Several in vitro and animal studies have pointed out that polyphenols decrease glucose absorption in the small intestine due to the inhibition of α-glucosidase, α-amylase, and sucrase in the gut mucosa, and they also limit their reabsorption in the kidney. Other mechanisms of action are related to peripheral tissues. That includes inhibition of gluconeogenesis, adrenergic stimulation of glucose uptake, and stimulation of insulin release by pancreatic β-cells [36].

The main limitation of the present study is the cross-sectional design, which does not allow us to assess causality, but also the estimation of polyphenol intake through FFQs, so bioavailability could not be considered. Furthermore, as in all epidemiological studies, residual confounding cannot be excluded. Finally, these results might not be generalizable to other populations. Our study has also several strengths, including the big sample size, the blinded assessment of the endpoint, the multicenter design, and the comprehensive data on dietary intake and risk factors of T2D and other potential confounders.

Many research studies endorse the role of polyphenols in modulating the risk of diseases. However, there are many issues to deal with when assessing these effects such as their wide variability of structures, the difficulty of analyzing polyphenol content in foods, the interactions between them, other food components and food matrices, and the complexity of the human body [37].

Authors should discuss the results and how they can be interpreted in the perspective of previous studies and of the working hypotheses. The findings and their implications should be discussed in the broadest context possible. Future research directions may also be highlighted.

## 5. Conclusions

We can conclude that high intakes of some polyphenols are inversely associated with the prevalence of T2D in older adults with metabolic syndrome, these relationships being more especially observed in overweight subjects than in obese ones. However, hydroxycinnamic acids are directly associated with T2D. In that sense, randomized controlled trials would be useful to confirm the promising benefits of polyphenols for the prevention and management of T2D.

## Figures and Tables

**Table 1:** Baseline characteristics by quartiles of energy-adjusted polyphenol intake (*n* = 6633).

Characteristics	Quartile 1	Quartile 2	Quartile 3	Quartile 4	*p*-Value
*n*	1659	1658	1658	1658	
Total polyphenols (mg/day), median (min–max)	561 (73–662)	736 (663–812)	896 (813–994)	1146 (995–3377)	
Sex, males, n (%)	775 (46.7)	871 (47.5)	775 (53.3)	679 (59.1)	<0.001
Age (years)	64.9 ± 4.9	65.0 ± 4.8	65.0 ± 4.9	65.1 ± 5.0	0.65
Body mass index (kg/m^2^)	32.6 ± 3.5	32.6 ± 3.5	32.6 ± 3.5	32.4 ± 3.3	0.07
Diabetic, n (%)	489 (29.5)	523 (31.5)	519 (31.3)	511 (30.8)	0.57
Insulin, n (%)	82 (4.9)	88 (5.3)	76 (4.6)	68 (4.1)	0.40
Metformin, n (%)	356 (21.5)	405 (24.4)	394 (23.8)	389 (23.5)	0.21
Other glucose lowering medication, n (%)	306 (18.4)	342 (20.6)	341 (20.6)	336 (20.3)	0.35
Fasting glucose (mg/dL)	113 ± 29	113 ± 29	114 ± 31	113 ± 28	0.59
Glycated hemoglobin (mmol/L)	6.1 ± 0.9	6.1 ± 0.8	6.1 ± 1.0	6.1 ± 0.8	0.54
Physical activity, (METS·min/week)	2252 ± 2216	2434 ± 2249	2437 ± 2275	2733 ± 2435	<0.001
Smoking status, n (%)					
Smoker	231 (13.9)	235 (14.2)	242 (14.6)	258 (15.6)	<0.001
Former, >1 year	623 (37.6)	642 (38.7)	709 (42.8)	762 (46.0)	
Never	804 (48.5)	780 (47.0)	706 (42.6)	638 (38.5)	
Education level, n (%)					
High	308 (18.6)	319 (19.2)	363 (21.9)	464 (28.0)	<0.001
Medium	484 (29.2)	467 (28.2)	485 (29.3)	477 (28.8)	
Low	867 (52.3)	872 (52.6)	810 (48.8)	717 (43.2)	

Values are frequencies and percentages for categorical variables or means ± SD for continuous variables, except for polyphenol intake which is represented by medians (min–max). *p*-values were calculated by chi-squared tests for categorical variables and ANOVA for continuous variables.

**Table 2:** Main dietary nutrient and food consumption according to quartiles of energy-adjusted polyphenol intake at baseline (*n* = 6633).

Nutrients and Foods	Quartile 1	Quartile 2	Quartile 3	Quartile 4	*p*-Value
*n*	1659	1658	1658	1658	
Total energy (kJ/day) (kcal/day)	10272 ± 2293 (2454 ± 548)	9481 ± 2256 (2265 ± 539)	9670 ± 2293 (2310 ± 548)	10180 ± 2294 (2432 ± 548)	<0.001
Carbohydrates (g/day)	246 ± 75	229 ± 70	237 ± 71	251 ± 73	<0.001
Fiber (g/day)	23 ± 7	25 ± 8	27 ± 8	30 ± 10	<0.001
Proteins (g/day)	100 ± 22	95 ± 22	96 ± 22	99 ± 22	<0.001
MUFAs (g/day)	57 ± 16	52 ± 16	52 ± 16	54 ± 16	<0.001
PUFAs (g/day)	18 ± 7	16 ± 6	16 ± 6	17 ± 6	<0.001
SFAs (g/day)	28 ± 9	25 ± 8	25 ± 8	26 ± 9	<0.001
Alcohol (g/day)	10 ± 14	10 ± 14	11 ± 14	14 ± 17	<0.001
17-point MedDiet score	7.7 ± 2.5	8.5 ± 2.6	8.7 ± 2.6	9.2 ± 2.7	<0.001
Food items (g/day)					
Vegetables	299 ± 125	319 ± 129	336 ± 134	357 ± 160	<0.001
Fruits	262 ± 146	324 ± 160	381 ± 181	470 ± 261	<0.001
Legumes	21 ± 11	21 ± 11	20 ± 11	21 ± 12	0.23
Cereals	164 ± 83	144 ± 74	147 ± 77	146 ± 77	<0.001
Dairy	358 ± 205	338 ± 193	341 ± 196	347 ± 211	0.02
Meat	160 ± 62	145 ± 58	143 ± 56	143 ± 56	<0.001
Olive oil	43 ± 18	39 ± 17	39 ± 16	39 ± 16	<0.001
Fish	101 ± 50	101 ± 48	102 ± 45	105 ± 47	0.05
Nuts	13 ± 16	14 ± 17	15 ± 17	17 ± 19	<0.001
Cookies, pastries, and sweets	27 ± 31	23 ± 28	25 ± 28	32 ± 32	<0.001
Sugar	8 ± 13	7 ± 11	6 ± 11	6 ± 12	<0.001
Soft drinks	31 ± 82	20 ± 56	20 ± 62	16 ± 51	<0.001

Values are means ± SD. *p*-values were calculated by ANOVA tests. Monounsaturated fatty acids, MUFAs; polyunsaturated fatty acids, PUFAs; saturated fatty acids, SFAs; Mediterranean diet, MedDiet.

**Table 3:** Prevalence ratio (PR) and confidence interval (CI) for prevalence of T2D and groups of energy-adjusted polyphenol intake by sex and BMI.

		Men						Women					
		Overweight (BMI < 30)			Obese (BMI ≥ 30)			Overweight (BMI < 30)			Obese (BMI ≥ 30)		
		*n* = 971 (314 cases, 32%)			*n* = 2453 (828 cases, 34%)			*n* = 802 (194 cases, 24%)			*n* = 2407 (706 cases, 29%)		
		Q4 vs. Q1	*p*-value	*p*-trend	Q4 vs. Q1	*p*-value	*p*-trend	Q4 vs. Q1	*p*-value	*p*-trend	Q4 vs. Q1	*p*-value	*p*-trend
Proanthocyanidins	Mean intake, mg/day	458.9 vs. 63.5			434.2 vs. 53.1			435.3 vs. 57.9			415.3 vs. 51.4		
	Cases	69 vs. 87			179 vs. 188			33 vs. 54			153 vs. 190		
	PR (CI)—unadjusted	0.81 (0.62–1.05)	0.11	0.18	0.96 (0.67–1.14)	0.67	0.79	0.62 (0.42–0.91)	0.02	0.01	0.81 (0.67–0.97)	0.02	0.02
	PR (CI)—model 1	0.81 (0.63–1.05)	0.12	0.20	0.98 (0.83–1.17)	0.83	0.95	0.64 (0.43–0.94)	0.02	0.01	0.82 (0.68–0.98)	0.03	0.03
	PR (CI)—model 2	0.75 (0.59–0.95)	0.02	0.04	0.93 (0.79–1.11)	0.43	0.48	0.51 (0.34–0.76)	0.001	<0.001	0.79 (0.66–0.95)	0.01	0.01
Flavanones	Mean intake, mg/day	185.3 vs. 16.6			180.4 vs. 16.8			179.8 vs. 17.6			183.6 vs. 15.3		
	Cases	90 vs. 74			211 vs. 203			48 vs. 53			179 vs. 184		
	PR (CI)—unadjusted	1.22 (0.95–1.57)	0.11	0.37	1.04 (0.89–1.22)	0.59	0.47	0.92 (0.66–1.30)	0.65	0.67	0.98 (0.82–1.16)	0.80	0.47
	PR (CI)—model 1	1.26 (0.98–1.62)	0.07	0.26	1.05 (0.90–1.23)	0.54	0.43	0.94 (0.67–1.33)	0.74	0.72	0.96 (0.81–1.14)	0.67	0.35
	PR (CI)—model 2	1.05 (0.84–1.32)	0.66	0.86	0.98 (0.85–1.15)	0.84	0.93	0.82 (0.58–1.16)	0.26	0.24	0.92 (0.78–1.09)	0.35	0.12
Flavones	Mean intake, mg/day	126.4 vs. 29.7			131.2 vs. 30.3			131.8 vs. 32.0			139.6 vs. 31.4		
	Cases	92 vs. 65			219 vs. 183			49 vs. 45			181 vs. 157		
	PR (CI)—unadjusted	1.41 (1.08–1.83)	0.01	0.004	1.21 (1.03–1.42)	0.02	0.01	1.11 (0.78–1.59)	0.54	0.87	1.16 (0.96–1.39)	0.12	0.13
	PR (CI)—model 1	1.42 (1.09–1.86)	0.01	0.003	1.22 (1.03–1.43)	0.02	0.009	1.16 (0.81–1.65)	0.43	0.69	1.18 (0.98–1.41)	0.08	0.08
	PR (CI)—model 2	1.00 (0.78–1.29)	0.99	0.65	0.99 (0.84–1.16)	0.90	0.90	0.94 (0.66–1.34)	0.73	0.50	1.08 (0.90–1.31)	0.39	0.35
Flavonols	Mean intake, mg/day	84.1 vs. 29.4			83.2 vs. 29.1			81.6 vs. 32.0			83.6 vs. 29.9		
	Cases	87 vs. 73			228 vs. 201			53 vs. 44			184 vs. 179		
	PR (CI)—unadjusted	1.24 (0.96–1.60)	0.11	0.06	1.13 (0.97–1.32)	0.11	0.18	1.22 (0.86–1.73)	0.26	0.14	1.03 (0.87–1.23)	0.72	0.54
	PR (CI)—model 1	1.25 (0.96–1.62)	0.10	0.05	1.13 (0.97–1.32)	0.12	0.21	1.27 (0.89–1.81)	0.11	0.09	1.09 (0.91–1.29)	0.34	0.25
	PR (CI)—model 2	0.97 (0.76–1.23)	0.79	0.95	0.96 (0.83–1.12)	0.62	0.45	1.16 (0.82–1.64)	0.40	0.19	1.05 (0.89–1.25)	0.57	0.50
Anthocyanidins	Mean intake, mg/day	94.9 vs. 13.9			97.6 vs. 13.3			82.0 vs. 11.8			87.5 vs. 10.1		
	Cases	67 vs. 73			224 vs. 174			47 vs. 47			172 vs. 185		
	PR (CI)—unadjusted	0.93 (0.70–1.23)	0.60	0.29	1.29 (1.10–1.52)	0.002	0.005	1.02 (0.72–1.45)	0.90	0.93	0.93 (0.79–1.11)	0.44	0.28
	PR (CI)—model 1	0.93 (0.70–1.23)	0.61	0.29	1.28 (1.09–1.51)	0.003	0.007	1.07 (0.75–1.54)	0.62	0.91	0.93 (0.79–1.11)	0.45	0.27
	PR (CI)—model 2	0.80 (0.62–1.05)	0.14	0.02	1.10 (0.94–1.29)	0.22	0.30	0.89 (0.63–1.26)	0.50	0.32	0.93 (0.78–1.10)	0.38	0.24
Catechins	Mean intake, mg /day	57.9 vs. 10.3			58.7 vs. 9.7			55.3 vs. 8.9			53.4 vs. 8.2		
	Cases	68 vs. 92			194 vs. 198			34 vs. 67			144 vs. 200		
	PR (CI)—unadjusted	0.75 (0.58–0.97)	0.03	0.03	0.99 (0.84–1.17)	0.92	0.69	0.51 (0.36–0.73)	0.001	<0.001	0.72 (0.60–0.86)	<0.001	<0.001
	PR (CI)—model 1	0.76 (0.58–0.98)	0.03	0.04	1.01 (0.86–1.19)	0.88	0.52	0.53 (0.37–0.77)	0.001	0.002	0.75 (0.62–0.90)	0.002	0.002
	PR (CI)—model 2	0.64 (0.51–0.81)	<0.001	<0.001	0.91 (0.77–1.06)	0.23	0.50	0.48 (0.33–0.69)	<0.001	<0.001	0.77 (0.64–0.92)	0.004	0.003
Hydroxycinnamic acids	Mean intake, mg/day	435.3 vs. 122.3			457.0 vs. 127.2			407.2 vs. 123.6			426.1 vs. 125.7		
	Cases	97 vs. 63			244 vs. 180			45 vs. 50			195 vs. 174		
	PR (CI)—unadjusted	1.54 (1.18–2.00)	0.001	0.003	1.36 (1.16–1.59)	<0.001	<0.001	1.13 (0.80–1.61)	0.49	0.46	1.13 (0.95–1.33)	0.17	0.19
	PR (CI)—model 1	1.52 (1.17–1.98)	0.002	0.005	1.35 (1.16–1.58)	<0.001	<0.001	1.14 (0.81–1.62)	0.45	0.44	1.12 (0.94–1.33)	0.20	0.20
	PR (CI)—model 2	1.62 (1.26–2.08)	<0.001	<0.001	1.43 (1.23–1.66)	<0.001	<0.001	1.15 (0.82–1.62)	0.41	0.40	1.16 (0.98–1.37)	0.08	0.09
Hydroxybenzoic acids	Mean intake, mg/day	30.7 vs. 6.5			31.7 vs. 5.8			24.2 vs. 5.2			22.3 vs. 4.2		
	Cases	72 vs. 78			208 vs. 197			38 vs. 59			149 vs. 191		
	PR (CI)—unadjusted	0.93 (0.71–1.22)	0.61	0.32	1.06 (0.90–1.24)	0.48	0.36	0.65 (0.45–0.93)	0.02	0.02	0.78 (0.65–0.94)	0.008	0.005
	PR (CI)—model 1	0.93 (0.71–1.22)	0.61	0.32	1.05 (0.89–1.23)	0.56	0.44	0.68 (0.47–0.97)	0.04	0.04	0.82 (0.68–0.99)	0.04	0.03
	PR (CI)—model 2	0.76 (0.60–0.97)	0.03	0.01	0.92 (0.79–1.07)	0.28	0.34	0.62 (0.43–0.89)	0.01	0.01	0.83 (0.69–1.00)	0.05	0.04
Stilbenes	Mean intake, mg/day	9.9 vs. 0.0			9.9 vs. 0.0			3.86 vs. 0.00			3.04 vs. 0.00		
	Cases	68 vs. 71			221 vs. 191			44 vs. 42			148 vs. 185		
	PR (CI)—unadjusted	0.99 (0.74–1.31)	0.93	0.50	1.16 (0.99–1.36)	0.07	0.09	1.04 (0.72–1.52)	0.74	0.83	0.81 (0.67–0.97)	0.03	0.02
	PR (CI)—model 1	0.99 (0.75–1.32)	0.97	0.54	1.16 (0.99–1.36)	0.07	0.09	1.10 (0.75–1.59)	0.55	0.63	0.83 (0.69–1.00)	0.05	0.04
	PR (CI)—model 2	0.80 (0.61–1.05)	0.12	0.03	0.99 (0.84–1.16)	0.78	0.97	0.92 (0.61–1.37)	0.68	0.63	0.86 (0.70–1.06)	0.15	0.12
Lignans	Mean intake, mg/day	2.20 vs. 1.00			2.22 vs. 1.00			2.12 vs. 1.33			2.15 vs. 1.00		
	Cases	71 vs. 75			221 vs. 207			44 vs. 47			156 vs. 197		
	PR (CI)—unadjusted	0.93 (0.71–1.22)	0.61	0.52	1.06 (0.91–1.24)	0.44	0.71	0.95 (0.66–1.36)	0.77	0.61	0.80 (0.67–0.95)	0.012	0.005
	PR (CI)—model 1	0.94 (0.66–1.24)	0.66	0.59	1.07 (0.91–1.25)	0.95	0.65	1.00 (0.69–1.45)	0.99	0.81	0.81 (0.68–0.97)	0.02	0.01
	PR (CI)—model 2	0.70 (0.54–0.90)	0.005	0.004	0.88 (0.76–1.03)	0.06	0.04	0.88 (0.61–1.26)	0.47	0.34	0.77 (0.64–0.92)	0.004	0.002

Model 1—adjusted by age, education (basic studies, medium, high), smoking (never, former, smoker), physical activity (quintiles). Model 2—adjusted by the variables used in model 1 plus total energy intake, consumption of refined cereals and animal products (from 17-point questionnaire) and of distilled beverages and liquors, sugar, soft drinks, cookies, and pastries (g/day).

## Supplementary Materials

The following are available online at https://www.mdpi.com/2076-3921/8/11/537/s1, List of the PREDIMED-Plus study investigators, Table S1: Energy-adjusted polyphenol intake (mg/day) according to sex and BMI groups (*n* = 6633), Table S2: Baseline characteristics by sex and BMI groups (*n* = 6633), Table S3: Main dietary nutrient and food composition according to sex and BMI groups (*n* = 6633).
