# Supplementary material for: Associations between Dietary Polyphenols and Type 2 Diabetes in a Cross-Sectional Analysis of the PREDIMED-Plus Trial: Role of Body Mass Index and Sex
Source: Antioxidants (Basel). 2019 Nov 8;8(11):537. doi: 10.3390/antiox8110537 (PMC6912253; doi:10.3390/antiox8110537)
Supplement: Supplementary file 1 [file antioxidants-08-00537-s001.pdf]

Supplementary material

List of PREDIMED-Plus study investigators

Steering Committee:
J. Salas-Salvadó (Coordinator), M.A. Martínez-González, M. Fitó. E. Ros, FJ. Tinahones, D. Corella and R. Estruch.

Executive Committee:
J. Salas-Salvadó, M.A. Martínez-González, D. Corella, M. Fitó, J. Vioque, D. Romaguera, J.A Martínez, J. Wärnberg, J. Lopez-Miranda, R. Estruch, A. Bueno-Cavanillas, Á.M. Alonso-Gómez, J.A. Tur, FJ. Tinahones, L. Serra-Majem, V. Martin, J. Lapetra, C. Vázquez, X. Pinto, J. Vidal, L. Daimiel, M. Delgado-Rodríguez, M.A. Rubio and E. Ros.

Dietary and Lifestyle Intervention Committee:
J. Salas-Salvadó (chair), M.A. Martínez-González, M. Fitó and R. Estruch;
Dietary Intervention: J. Salas-Salvadó (chair), N. Babio, E. Ros, A. Sánchez-Tainta;
Physical Exercise: M. Fitó (chair), H. Schröder, A. Marcos, D. Corella, J. Warnberg;
Behavioural support: R. Estruch (chair), F. Fernández-Aranda, C. Botella and J. Salas-Salvadó.

Clinical Event Ascertainment Committee:
F. Arós (Chair), M. Aldamiz, A. Alonso-Gómez, L. Forga, A. García-Layana, J. Portu, J. Timiraos, A. González-Pinto, I. Zorrilla, M. Martínez-Kareaga, P. Seoane.

Chair: Dr. Fernando Arós
Cardiology: Dr. Angel Alonso-Gómez; Dr. Fernando Arós
Neurology: Dr. Juan Timiraos
Internal Medicine: Dr. Mikel Aldamiz; Dr. Joseba Portu
Endocrinology: Dr. Lluis Forga
Ophthalmology: Dr. Alfredo García-Layana
Psychiatry: Dr. Ana González Pinto; Dr. Iñaki Zorrilla
Oncology: Dr. Mireia Martínez; Dr. Patricia Seoane

Support groups:

C. Botella, F. Fernandez-Aranda, R. Lamuela, A. Marcos, M.P. Portillo, E. Ros, G. Sáez, F. Arós, E. Gómez-Gracia

Rovira i Virgili University, Department of Biochemistry and Biotechnology, Human Nutrition Unit, University Hospital of Sant Joan de Reus, Pere Virgili Institute for Health Research, Reus, Spain: R. Pedret Llaberia, R. Gonzalez, R. Sagarra Álamo, F. París Palleja, J. Balsells, J.M. Roca, T. Basora Gallisa, J. Vizcaino, P. Llobet Alpizarte, C. Anguera Perpiñá, M. Llauradó Vernet, C. Caballero, M. Garcia Barco, M.D. Morán Martínez, J. García Rosselló, A. Del Pozo, C. Poblet Calaf, P. Arcelin Zabal, X. Floresví, M. Ciutat Benet, A. Palau Galindo, J.J. Cabré Vila, F. Dolz Andrés, M. Soler, M. Gracia Vidal, J. Vilalta J. Boj Casajuana, M. Ricard, F. Saiz, A. Isach, M. Sanchez Marin Martinez, E. Granado Font, C, Lucena Luque, C. Mestres Sola, M. Bulló, N. Babio, N. Becerra-Tomás, G. Mestres, J. Basora, G. Mena-Sánchez, L. Barrubés Piñol, M. Gil Segura, C. Papandreou, N. Rosique-Esteban, S. Chig, I. Abellán Cano, V. Ruiz García, A. Salas-Huetos, I. Paz-Graniel, L. Sánchez Niembro, P. Hernandez-Alonso, S. Canudas, L. Camacho-Barcia, J. García-Gavilán, A. Díaz-López.

Department of Preventive Medicine and Public Health, University of Navarra-Navarra Institute for Health Research (IdiSNA), Pamplona, Spain: M. Ruiz-Canela, E. Toledo, P. Buil-Cosiales, Z. Vázquez, C. Razquin, M. Bes-Rastrollo, A. Gea, A. Sanchez Tainta, B. SanJulian Aranguren, E. Goñi, L. Goñi, M.J. Cobo, A. Rico-Campa, F.J. Basterra Gortari, A. Garcia Arellano, J. Diez-Espino, O. Lecea-Juarez, J. Carlos Cenoz-Osinaga, I. Alvarez-Alvarez, M.C. Sayon-Orea, C.I. Fernandez-Lázaro, L. Ruiz-Estigarribia, J. Bartolome-Resano, A. Sola-Larraza (†), E. Lozano-Oloriz, B. Cano-Valles, S. Eguaras, E. Pascual Roquet-Jalmar, I. Galilea-Zabalza, H. Lancova, R. Ramallal, M.L. Garcia-Perez, V. Estremera-Urabayen, M.J. Ariz-Arnedo, C. Hijos-Larraz, C. Fernandez-Alfaro, B. Iñigo-Martinez, R. Villanueva Moreno, S. Martin-Almendros, L. Barandiaran-Bengoetxea, C. Fuertes-Goñi, A. Lezaun-Indurain, M.J. Guruchaga-Arcelus, O. Olmedo-Cruz, L. Escriche-Erviti, R. Ansorena-Ros, R. Sanmatin-Zabaleta, J. Apalategi-Lasa, J. Villanueva-Telleria, M.M. Hernández-Espinosa, L. Herrera-Valdez, L. Dorronsoro-Dorronsoro, Lourdes Echeverria-Lizarraga (†), J.A. Cabeza-Beunza, P. Fernández-Urretavizcaya, P. Gascó-García, C. Royo-Jimenez, J. Moran-Pí, F. Salazar-Fernández, F.J. Chasco-Ros, F. Cortés-Ugalde, J.J. Jurio-Burgui, P. Pascual-Pascual, A.I. Rodríguez-Ezpeleta, M. Esparza-Cáceres, C.

Arroyo-Azpa, M. Rodríguez-Sanz de Galdeano, T. Forcen-Alonso, M. Armendariz-Marcotegui, A. Brugos-Larumbe, A. Arillo, B. López-Aisa.

Department of Preventive Medicine, University of Valencia, University Jaume I, Conselleria de Sanitat de la Generalitat Valenciana, Valencia, Spain: J.I. González, J.V. Sorlí, O. Portolés, R. Fernández-Carrión, C. Ortega-Azorín, R. Barragán, E.M. Asensio, O. Coltell, R. Martínez-Lacruz, I. Giménez-Alba, C. Sáiz, R. Osma, E. Férriz, I. González-Monje, P. Guillém-Sáiz, F. Giménez-Fernández, L. Quiles, P. Carrasco, A. Carratalá-Calvo, C. Valero-Barceló, C. Mir, S. Sánchez-Navarro, J. Navas, I. González-Gallego, L. Bort-Llorca, L. Pérez-Ollero, M. Giner-Valero, R. Monfort-Sáez, J. Nadal-Sayol, V. Pascual-Fuster, M. Martínez-Pérez, C. Riera, M.V. Belda, A. Medina, E. Miralles, M.J. Ramírez-Esplugues, M. Rojo-Furió, G. Mattingley, M.A. Delgado, M.A. Pages, Y Riofrío, L. Abuomar, N. Blasco-Lafarga, R. Tosca, L. Lizán, A.M Valcarce, M.D. Medina, S. de Valcárcel, N. Tormo, O. Felipe-Román, S. Lafuente, E.I. Navío, G. Aldana, J.V. Crespo, J.L. Llosa, L. González-García, R. Raga-Marí.

Cardiovascular Risk and Nutrition Research Group, Endocrinology Service, Neurosciences Programme, Clinical Research Unit at the Hospital del Mar Medical Research Institute (IMIM), Barcelona. Medicine Departament, Universitat Autònoma de Barcelona, Barcelona, Spain: M. Fitó, O. Castañer, M.A. Muñoz, M.D. Zomeño, A. Hernaéz, L. Torres, M. Quifer, R. Llimona, G Freixer, KA. Pérez-Vega, M. Farràs, R. Elosua, J. Vila, I. Subirana, S. Pérez, A. Goday, J.J. Chillaron Jordan, J.A. Flores Lerroux, D. Benaiges Boix, G. Llauradó, M. Farré, E. Menoyo, A. Aldea-Perona, M. Pérez-Otero, D. Muñoz-Aguayo, S. Gaixas, G. Blanchart, A. Sanllorente, M. Soria, J. Valussi, A. Cuenca, L. Forcano, A. Pastor, A. Boronat, S. Tello, M. Cabañero, L. Franco, H. Schröder, R. De la Torre, C. Medrano, J. Bayó, M.T. García, V. Robledo, P. Babi, E. Canals, N. Soldevila, L. Carrés, C. Roca, M.S. Comas, G. Gasulla, X. Herraiz, A. Martínez, E. Vinyoles, J.M. Verdú, M. Masague Aguade, E. Baltasar Massip, M. López Grau, M. Mengual, V. Moldon, M. Vila Vergaz, R. Cabanes Gómez, Ciurana, M. Gili Riu, A. Palomeras Vidal, F Peñas F, A Raya, M.A. Sebastian, M. Valls, J. Guerrero, M. Marne, E. Minguella, M. Montenegro, A. Sala, M.R. Senan, N. Talens, N. Vera.

Nutritional Epidemiology Unit, Miguel Hernandez University, ISABIAL-FISABIO, Alicante, Spain: J. Vioque, M. García-de-la-Hera, E.M. Navarrete-Muñoz, S. Gonzalez-Palacios, L. Torres-Collado, L. Compañ-Gabucio, A. Oncina-Canovas, L. Notario-Barandiaran, D. Orozco-Beltran, S. Pertusa Martínez, A. Asensio, I. Candela-García, J.M. Zazo, C. Gisbert Sellés, N. Fernándis-Brufal, J. Román Maciá, C. Sánchez Botella, M. García Muñoz, C. Barceló, M.C. Altozano-Rodado, N. Iranzo García, M.C. Martínez Vergara, M.A. Sempere Pascual, S.J. Miralles Gisbert, A. González Botella, C.M. López García, R. Valls Enguix, N. Gómez Bellvert, I. López Aguilera, R. Lloret Macián, A. Pastor Morell, E. Alonso Bartolomé, J.J. Ballester Baixauli, M.T. Cano Sánchez, B.E. Ayús Rojo, E.P. Cases Pérez, C. Tercero Maciá, L.A. Mira Castejón, I.A. García García, M. Jordá Ballesta, C. Pastor Polo, E. Puig Agulló

Hospital Son Espases (HUSE) and Institute for Health Research Illes Balears (IdISBa), Palma de Mallorca, Spain: M. Fiol, M. Moñino, A. Colom, J. Konieczna, M. Morey, R. Zamanillo, A.M. Galmés-Panadés, V. Pereira, M.A. Martín, A. Yáñez, J. Llobera, J. Ripoll, R. Prieto, F. Grases, A. Costa, C. Fernández-Palomeque, E. Fortuny, M. Noris, S. Munuera, F. Tomás, F. Fiol, A. Jover, J.M. Janer, C. Vallespir, I. Mattei, N. Feuerbach, M. del Mar Sureda, S. Vega, L. Quintana, A. Fiol, M. Amador, S. González, J. Coll, A. Moyá, T. Piqué Sistac, M.D. Sanmartín Fernández, M.C. Piña Valls, M.A. Llorente San Martín, J. Pou Bordoy.

Department of Nutrition, Food Sciences, and Physiology, Center for Nutrition Research, University of Navarra, Pamplona, Spain: I. Abete, I. Cantero, C. Cristobo, I. Ibero-Baraibar, M. Zulet, J. Ágreda-Peiró, M.D. Lezáun-Burgui, N. Goñi-Ruiz, R. Bartolomé-Resano, E. Cano-Cáceres, T. Elcarte-López, E. Echarte-Osacain, B. Pérez-Sanz, I. Blanco-Platero, A. Andueza- Azcárate, A. Gimeno-Aznar, E. Ursúa-Sesma, B. Ojeda-Bilbao, J. Martinez-Jarauta, L. Ugalde-Sarasa, B. Rípodas-Echarte, M.V. Güeto-Rubio, C. Napal-Lecumberri, MD Martínez-Mazo, E Arina-Vergara, A. Parra-Osés, F. Artal-Moneva, F. Bárcena-Amigo, F. Calle-Irastoza, J. Abad-Vicente, J.I. Armendáriz-Artola, P. Iñigo-Cibrian, J. Escribano-Jarauta, J. Ulibarri-delportillo, B. Churio-Beraza, Y. Monzón-Martínez, E. Madoz-Zubillaga, C. Arroniz.

University of Málaga and Institute of Biomedical Research in Malaga (IBIMA), Málaga, Spain: F.J. Barón-López, J.C. Fernández García, N. Pérez-Farinós, N. Moreno-Morales, M. del C. Rodríguez-Martínez, J. Pérez-López, J.C. Benavente-Marín, E. Crespo Oliva, E. Contreras Fernández, F.J. Carmona González, R. Carabaño Moral, S. Torres Moreno, M.V. Martín Ruíz, M. Alcalá Cornide, V. Fuentes Gómez.

Lipids and Atherosclerosis Unit, Department of Internal Medicine, Maimonides Biomedical Research Institute of Cordoba (IMIBIC), Reina Sofia University Hospital, University of Cordoba, Cordoba, Spain: J. López-Miranda, A. Garcia-Rios, J. Criado García, A.I. Jiménez Morales, A. Ortiz Morales, J.D. Torres Peña, F.J. Gómez Delgado, J.F. Alcalá, A. León Acuña, A.P. Arenas Larriva, F. Rodríguez Cantalejo, J. Caballero Villaraso, I. Nieto Eugenio, P. Coronado Carvajal, M.C del Campo Molina, P.J. Peña Orihuela, I. Perez Corral, G. Quintana Navarro.

Department of Internal Medicine, Institut d'Investigacions Biomèdiques August Pi i Sunyer (IDIBAPS), Hospital Clínic, University of Barcelona, Barcelona, Spain: R. Casas, M. Domenech, C. Viñas, S. Castro-Barquero, A.M. Ruiz-León, R. Losno, L. Tarés, A. Jordán, R. Soriano, M. Camafort, C. Sierra, E. Sacanella, A. Sala-Vila, J. M. Cots, I. Sarroca, M. García, N. Bermúdez, A. Pérez, I. Duaso, A. de la Arada, R. Hernández, C. Simón, M.A. de la Poza, I. Gil, M. Vila, C. Iglesias, N. Assens, M. Amatller, LL. Rams, T. Benet, G. Fernández, J. Teruel, A. Azorin, M. Cubells, D. López, J.M. Llovet, M.L. Gómez, P. Climente, L. de Paula, J. Soto, C. Carbonell, C. Llor, X. Abat, A. Cama, M. Fortuny, C. Domingo, A. I. Liberal, T. Martínez, E. Yañez, M. J. Nieto, A. Pérez, E. Lloret, C. Carrazoni, A. M. Belles, C. Olmos, M. Ramentol, M. J. Capell, R. Casas, I. Giner, A. Muñoz, R. Martín, E. Moron, A. Bonillo, G. Sánchez, C. Calbó, J. Pous, M. Massip, Y. García, M.C. Massagué, R. Ibañez, J. Llaona, T. Vidal, N. Vizcay, E. Segura, C. Galindo, M. Moreno, M. Caubet, J. Altirriba, G. Fluxà, P. Toribio, E. Torrent, J. J. Anton, A. Viaplana, G. Vieytes, N. Duch, A. Pereira, M. A. Moreno, A. Pérez, E. Sant, J. Gené, H. Calvillo, F. Pont, M. Puig, M. Casasayas, A. Garrich, E. Senar, A. Martínez, I. Boix, E. Sequeira, V. Aragunde, S. Riera, M. Salgado, M. Fuentes, E. Martín, A. Ubieto, F. Pallarés, C. Sala, A. Abilla, S. Moreno, E. Mayor, T. Colom, A. Gaspar, A. Gómez, L. Palacios, R. Garrigosa.

Department of Preventive Medicine and Public Health, University of Granada, Granada, Spain: L. García Molina, B. Riquelme Gallego, N. Cano Ibañez, A. Maldonado Calvo, A. López Maldonado, E.M. Garrido, A. Baena Dominguez, F. García Jiménez, E. Thomas Carazo, A. Jesús Turnes González, F. González Jiménez, F. Padilla Ruiz, J. Machado Santiago, M.D. Martínez Bellón, A. Pueyos Sánchez, L. Arribas Mir, R. Rodríguez Tapioles, F. Dorador Atienza, L. Baena Camus, C. Osorio Martos, D. Rueda Lozano, M. López Alcázar, F. Ramos Díaz, M. Cruz Rosales Sierra, P. Alguacil Cubero, A. López Rodriguez, F. Guerrero García, J. Tormo Molina, F. Ruiz Rodríguez.

Bioaraba Health Research Institute, Cardiovascular, Respiratory and Metabolic Area; Osakidetza Basque Health Service, Araba University Hospital; University of the Basque Country UPV/EHU, Vitoria-Gasteiz, Spain: I. Salaverria, A. Alonso-Gómez, M.C. Belló, L. Tojal, L. Goicolea, C. Sorto, A Goikoetxea, A. Casi Casanellas, M.L. Arnal Otero, J. Ortueta Martínez De Arbulo, J. Vinagre Morgado, J. Romeo Ollora, J. Urraca, M.I. Sarriegui Carrera, F.J. Toribio, E. Magán, A. Rodríguez, S. Castro Madrid, M.T. Gómez Merino, M. Rodríguez Jiménez, M. Gutiérrez Jodra, B. López Alonso, J. Iturralde Iriso, C. Pascual Romero, A. Izquierdo De La Guerra.

Research Group on Community Nutrition & Oxidative Stress, University of Balearic Islands, Palma de Mallorca, Spain: M. Abbate, E. Angullo, E. Argelich, M.M. Bibiloni, C. Bouzas, X. Capó, S. Carreres, L. Gallardo, J.M. Gámez, B. García, C. García, A. Julibert, C. Gómez, I. Llompart, A. Martorell, C.M. Mascaró, D. Mateos, M, Monserrat, S. Montemayor, A. Pons, A. Pouso, J. Ramos, V. Ramos, T. Ripoll, T. Rodríguez, L. Sanz, A. Sureda, S. Tejada, L. Ugarriza.

Virgen de la Victoria Hospital, University of Málaga, Málaga, Spain: M.R. Bernal López, M. Macías González, J. Ruiz Nava, J.C. Fernández García, A. Muñoz Garach, A. Vilches Pérez, A. González Banderas, A.V. Alarcón-Martín, M. García Ruiz de Mier, J. Alcaide Torres, A. Vargas Candela, M. León Fernández, R. Hernández Robles, S. Santamaría Fernández, J.M. Marín.

University of Las Palmas de Gran Canaria, Las Palmas, Spain: J. Álvarez-Pérez, E.M. Díaz Benítez, F. Díaz-Collado, A. Sánchez-Villegas, J. Pérez-Cabrera, L.T. Casañas-Quintana, R.B. García-Guerra, I. Bautista-Castaño, C. Ruano-Rodríguez, F. Sarmiento de la Fe, J.A. García-Pastor, B. Macías-Gutiérrez, I. Falcón-Sanabria, C. Simón-García, A.J. Santana-Santana, J.B. Álvarez-Álvarez, B.V. Díaz-González, J.M. Castillo Anzalas, R.E. Sosa-Also, J. Medina-Ponce.

Biomedicine Institute (IBIOMED); University of León, and Primary Health Care Management of León (Sacyl), León, Spain: S. Abajo Olea, A. Adlbi Sibai, M. Ajenjo, L. Álvarez, E. Carriedo Ule, N. Cubelos, V. Dávila-Batista, M. Escobar Fernández, P. Farias Cardoso, T. Fernández-Villa, J.I. Ferradal García, J.P. Fernández Vázquez, F. González Rivero, A. Marcos-Delgado, V. Martín, C. Onrrubia Baticón, M. Lavinia Popescu, J.I. López Gil, J. López de la Iglesia, A.J. Molina, M. Piró, S. Reguero Celada, M. Rodríguez Bul, M. Rubín-García, A.A. Torres Serna, F. Vitelli-Storelli.

Department of Family Medicine, Distrito Sanitario Atención Primaria Sevilla, Sevilla, Spain: J.M. Santos-Lozano, L. Miró-Moriano, C. Domínguez-Espinaco, S. Vaquero-Díaz, F.J. García-Corte, A. Santos-Calonge, C. Toro-Cortés, N. Pelegrina-López, V. Urbano-Fernández, M. Ortega-Calvo, J. Lozano-Rodríguez, I. Rivera-Benítez, M. Caballero-Valderrama, P. Iglesias-Bonilla, P. Román-Torres, Y. Corchado-Albalat, L. Mellado-Martín.

Department of Endocrinology and Nutrition, Hospital Fundación Jimenez Díaz. Instituto de Investigaciones Biomédicas IISFJD. University Autonoma, Madrid, Spain: A.I. de Cos, S. Gutierrez, S. Artola, A. Galdon, I. Gonzalo.

Lipids and Vascular Risk Unit, Internal Medicine, University Hospital of Bellvitge- IDIBELL, Hospitalet de Llobregat, Barcelona, Spain: X. Pintó, A. Galera, M. Gimenez-Gracia, E. de la Cruz, R. Figueras, M. Poch, R. Freixedas, F. Trias, I. Sarasa, M. Fanlo-Maresma, H. Lafuente, M. Liceran, A. Rodriguez-Sanchez, C. Pallarols, E. Gómez-Sanchez, V. Esteve-Luque, J. Monedero, X. Corbella, E. Corbella.

Department of Endocrinology, IDIBAPS, Hospital Clinic, University of Barcelona, Barcelona, Spain: A. Altés, I. Vinagre, C. Mestre, J. Viaplana, M. Serra, J. Vera, T. Freitas, E. Ortega, I. Pla, R. Olbeyra.

Nutritional Genomics and Epigenomics Group, Institute IMDEA-Food, CEI UAM+CSIC, Madrid, Spain: J.M. Ordovás, V. Micó, L. Berninches, M.J. Concejo, J. Muñoz, M. Adrián, Y. de la Fuente, C. Albertos, E. Villahoz, M.L. Cornejo, C. Cuesta. Montero A., Valdés, MC.

Division of Preventive Medicine, University of Jaén, Jaén, Spain: J.J. Gaforio, S. Moraleda, N. Liétor, J.I. Peis, T. Ureña, M. Rueda, M.I. Ballesta.

Department of Endocrinology and Nutrition, Hospital Clínico San Carlos, Instituto de Investigación Sanitaria del Hospital Clínico San Carlos (IdISSC), Madrid, España (Spain para internacionales: C. Moreno Lopera, C. Aragoneses Isabel, M.A. Sirur Flores, M. Ceballos de Diego, T. Bescos Cáceres, Y. Peña Cereceda, M. Martínez Abad, R. Cabrera Vélez, M. González Cerrajero, M.A. Rubio Herrera, M. Torrego Ellacuría, A. Barabash Bustelo, M. Ortiz Ramos, A. Larrad Sainz.

Supplementary table 1. Energy-adjusted polyphenol intake (mg/day) according to sex and BMI groups (n=6633)

	Men			Women			
	Overweight (BMI<30)	Obese (BMI≥30)	P value[1]	Overweight (BMI<30)	Obese (BMI≥30)	P value[2]	P value[3]
N	971	2453		802	2407		
Total polyphenols	875.3±276.3	872.4±291.9	0.78	818.3±255.7	816.0±259.8	0.83	<0.001
Total flavonoids	509.1±230.3	490.6±235.9	0.04	495.7±214.5	484.5±220.9	0.21	0.13
Proanthocyanidins	222.1±174.1	203.4±182.3	0.01	209±169.9	197.9±167.8	0.10	0.06
Flavanones	85.8±79	85.0±73.8	0.78	88.1±73.6	87.2±75.9	0.79	0.23
Flavones	68.8±43.2	70.2±45.4	0.41	73.6±42.0	75.9±46.1	0.21	<0.001
Flavonols	53.8±22.1	53.5±22.1	0.68	54.1±19.7	53.8±21.6	0.68	0.60
Anthocyanidins	46.8±34.3	47.4±35.9	0.63	39.5±31.2	40.0±38.8	0.75	<0.001
Catechins	30.1±20.3	29.4±22.4	0.43	27±22.1	25.7±21.2	0.14	<0.001
Total phenolic acids	285.2±129.9	298.3±135.6	0.01	258.3±116.2	267.4±118.3	0.06	<0.001
HCA	260.4±129.4	273.5±134	0.01	248.9±115.8	259.4±118.2	0.03	<0.001
HBA	17.2±9.9	17.1±10.7	0.83	13.3±8.0	12.0±7.5	<0.001	<0.001
Stilbenes	3.40±4.66	3.40±4.76	0.97	1.18±2.18	0.94±1.93	0.003	<0.001
Lignans	1.56±0.46	1.57±0.53	0.50	1.51±0.47	1.51±0.50	0.91	<0.001

Values are means±SD. P-values were calculated by ANOVA tests (1) between BMI groups in men, (2) between BMI groups in women, (3) between men and women. HCA: Hydroxycinnamic acids, HBA: Hydroxybenzoic acids

Supplementary table 2. Baseline characteristics by sex and BMI groups (n=6633)

	Men			Women			
	Overweight (BMI<30)	Obese (BMI≥30)	P value[1]	Overweight (BMI<30)	Obese (BMI≥30)	P value[2]	P value[3]
N	971	2453		802	2407		
Age (years)	64.3±5.3	63.6±5.3	<0.001	66.3±4.1	66.3±4.0	0.88	<0.001
Body Mass Index (Kg/m^2)	28.6±0.9	33.7±2.7	<0.001	28.4±1.0	34.3±2.9	<0.001	<0.001
Diabetic, n (%)	314 (32.3)	828 (33.8)	0.43	194 (24.2)	706 (29.3)	0.01	<0.001
Glucose lowering medication, n (%)	211 (21.7)	547 (22.3)	0.72	126 (15.7)	441 (18.3)	0.09	<0.001
Insulin, n (%)	37 (3.8)	131 (5.3)	0.06	28 (3.5)	118 (4.9)	0.10	0.49
Metformin, n (%)	251 (25.9)	637 (26.0)	0.94	134 (16.7)	522 (21.7)	0.002	<0.001
Fasting glucosa (n=6,531), mg/dL	114±31.5	116.4±30.7	0.04	107.1±22.7	112.5±27.8	<0.001	<0.001
Glycated hemoglobin (n=6,037), mmol/L	6.1±1.0	6.1±0.9	0.31	6.0±0.7	6.1±0.8	0.001	0.15
Physical activity, METS·min/week	3135±2537	2753±2607	<0.001	2272±1897	1962±1830	<0.001	<0.001
Smoking status, n (%)			0.08			0.01	<0.001
Smokers	209 (21.5)	458 (18.7)		96 (12.0)	203 (8.4)		
Former, >1 year	538 (55.5)	1455 (59.3)		180 (22.4)	563 (23.4)		
Never	224 (23.0)	540 (22.0)		526 (65.6)	1641 (68.2)		
Education level, n (%)			0.02			<0.001	<0.001
High	306 (31.5)	670 (27.3)		154 (19.2)	324 (13.4)		
Medium	321 (33.1)	797 (32.5)		213 (26.6)	582 (24.2)		
Low	344 (35.4)	986 (40.2)		435 (54.2)	1501 (62.4)		

Values are frequencies and percentages for categorical variables or means±SD for continuous variable. P-values were calculated by Chi^2 tests for categorical variables and ANOVA for numerical, (1) between BMI groups in men, (2) between BMI groups in women, (3) between men and women.

Supplementary table 3. Main dietary nutrient and food composition according to sex and BMI groups (n=6633)

	Men			Women			
	Overweight (BMI<30)	Obese (BMI≥30)	P value[1]	Overweight (BMI<30)	Obese (BMI≥30)	P value[2]	P value[3]
N	971	2453		802	2407		
Total energy (KJ/d) (Kcal/d)	10494±2273 (2507±543)	10536±2369 (2517±566)	0.62	9276±1942 (2216±464)	9222±2114 (2203±505)	0.50	<0.001
Carbohydrates (g/d)	256±76	252±76	0.13	228±64	228±67	0.84	<0.001
Fiber (g/d)	26±9	25±9	0.44	27±9	27±9	0.15	<0.001
Proteins (g/d)	98±22	100±23	0.08	96±20	96±22	0.88	<0.001
MUFA (g/d)	55.7±15.8	56.7±16.4	0.11	51.3±15.9	50.8±15.9	0.47	<0.001
PUFA (g/d)	17.6±6.4	17.6±6.6	0.98	16.4±6.1	15.6±6.0	0.002	<0.001
SFA (g/d)	27.6±8.4	28.0±8.7	0.17	24.4±7.4	24.5±8.0	0.65	<0.001
Alcohol (g/d)	16±16	17±18	0.12	5±8	4±7	0.03	<0.001
17-points MedDiet score	8.3±2.6	8.1±2.7	0.13	9.2±2.7	8.8±2.6	<0.001	<0.001
Food items, g/day							
Vegetables	313±135	311±135	0.66	347±138	345±143	0.77	<0.001
Fruits	345±205	338±197	0.34	382±201	379±216	0.69	<0.001
Legumes	21±11	21±11	0.78	21±11	20±11	0.53	0.06
Cereals	162±83	162±83	0.96	136±69	138±70	0.35	<0.001
Dairy	325±196	326±197	0.97	376±209	365±202	0.18	<0.001
Meat	148±59	157±61	<0.001	137±54	141±56	0.07	<0.001
Olive oil	40±17	41±17	0.46	39±17	39±17	0.53	<0.001
Fish	102±46	100±49	0.28	104±45	104±48	0.82	0.001
Nuts	16±16	15±18	0.13	17±19	14±17	<0.001	0.24
Cookies, pastries and sweets	30±34	28±31	0.33	24±24	25±28	0.33	<0.001
Sugar	9±14	8±13	0.04	5±10	5±9	0.11	<0.001
Soft drinks	23±60	27±78	0.19	18±48	17±53	0.92	<0.001

Values are means±SD. P-values were calculated by ANOVA tests (1) between BMI groups in men, (2) between BMI groups in women, (3) between men and women.
MUFA Monounsaturated Fatty Acids, PUFA Polyunsaturated Fatty Acids, SFA Saturated Fatty Acids, MedDiet Mediterranean Diet.
